# Pacientes com baixo peso e baixa densidade óssea correm maior risco de fratura intraoperatória durante a hemiartroplastia

**DOI:** 10.1055/s-0045-1814126

**Published:** 2025-12-22

**Authors:** Jun Takeuchi

**Affiliations:** 1 Departmento de Cirurgia Ortopédica, Hospital Yonemori, Yojirou, Cidade de Kagoshima, Kagoshima, Japão

**Keywords:** densidade mineral óssea, fatores de risco, fraturas do colo femoral, fraturas do fêmur, índice de massa corporal, body mass index, bone mineral density, femoral fractures, femoral neck fractures, risk factors

## Abstract

**Objetivo:**

Investigar se o índice de massa corporal (IMC) modifica a associação entre densidade mineral óssea (DMO) e risco de fratura intraoperatória em hemiartroplastia não cimentada.

**Métodos:**

Entre abril de 2020 e março de 2025, foram incluídos 588 pacientes que receberam hastes não cimentadas e foram submetidos a exames de raios X de dupla energia dentro de uma semana após a cirurgia. Os pacientes foram estratificados por IMC (≥ 18,5 kg/m ^2^ versus < 18,5 kg/m ^2^ ) e porcentagem média de adultos jovens (YAM, do inglês *young adult mean* ) (ponto de corte: 70%). O risco de fratura foi comparado entre os grupos. Análises de subgrupos, com base nos níveis de YAM e nos parâmetros operatórios, foram realizadas em 577 pacientes com dados completos.

**Resultados:**

Em pacientes com baixo peso (IMC < 18,5 kg/m ^2^ ), menor YAM associou-se a maior risco de fratura intraoperatória ( *p* = 0,128), enquanto nenhuma associação significativa foi observada em pacientes com IMC ≥ 18,5 kg/m ^2^ ( *p* = 0,80). As taxas de fratura aumentaram com menor YAM: 3,97% (≥ 70%), 5,24% (60–69%), 5,88% (50–59%), 6,58% (< 50%), embora esse aumento não tenha sido estatisticamente significativo. O IMC mais alto foi associado a maior tempo operatório e maior perda de sangue.

**Conclusão:**

O IMC modifica a associação entre DMO e risco de fratura intraoperatória. A avaliação pré-operatória do YAM pode ajudar a identificar pacientes com baixo peso e com maior risco durante a hemiartroplastia não cimentada. Nível de evidência III, estudo terapêutico.

## Abstract (en)

**Objective:**

To investigate whether body mass index (BMI) modifies the association be tween bone mineral density (BMD) and intraoperative fracture risk in cementless hemiarthroplasty.

**Methods:**

Between April 2020 and March 2025, 588 patients who received cementless stems and underwent dual-energy X-ray absorptiometry scans within one week postoperatively were included. Patients were stratified by BMI (≥ 18.5 kg/m ^2^ versus < 18.5 kg/m ^2^ ) and young adult mean (YAM) (cutoff: 70%). Fracture risk was compared across groups. Subgroup analyses based on YAM levels and operative parameters were performed in 577 patients with complete data.

**Results:**

In underweight patients (BMI < 18.5 kg/m ^2^ ), lower YAM was associated with higher intraoperative fracture risk ( *p* = 0.128), whereas no significant association was observed in patients with BMI ≥ 18.5 kg/m ^2^ ( *p* = 0.80). Fracture rates increased with lower YAM: 3.97% (≥ 70%), 5.24% (60–69%), 5.88% (50–59%), 6.58% (< 50%), though not statistically significant. Higher BMI was associated with longer operative time and greater blood loss.

**Conclusion:**

Body mass index modifies the association between BMD and intraoperative fracture risk. Preoperative YAM assessment may help identify underweight patients at higher risk during cementless hemiarthroplasty. Level of Evidence III, therapeutic study

## Introdução

As fraturas proximais do fêmur são lesões comuns em populações idosas, representando um grande ônus para a saúde pública em todo o mundo, devido ao envelhecimento da população. A artroplastia de quadril é frequentemente realizada para fraturas deslocadas do colo do fêmur, proporcionando resultados funcionais favoráveis e alívio da dor. No entanto, as fraturas periprotéticas intraoperatórias continuam sendo uma das principais complicações durante os procedimentos de artroplastia, particularmente em pacientes idosos e com osteoporose.

A densidade mineral óssea (DMO) é considerada um fator-chave no risco de fratura intraoperatória. A avaliação pré-operatória da DMO, frequentemente expressa como uma porcentagem média de adultos jovens ( *young adult mean* [YAM], em inglês), tem sido proposta como um parâmetro útil para avaliar a qualidade óssea em candidatos cirúrgicos. Além disso, o índice de massa corporal (IMC), que reflete o estado nutricional e as tendências sarcopênicas, também pode desempenhar papel importante na fragilidade óssea e na suscetibilidade à fratura.

Embora tanto a DMO quanto o IMC sejam reconhecidos como contribuintes importantes para a resistência óssea, estudos limitados exploraram a interação potencial entre esses fatores no contexto de fraturas periprotéticas intraoperatórias. Compreender como o IMC modifica a associação entre a DMO e o risco de fratura intraoperatória pode oferecer perspectivas úteis para a estratificação do risco cirúrgico e a otimização pré-operatória.

O objetivo deste estudo foi investigar se o IMC modifica o valor preditivo da DMO para fraturas periprotéticas intraoperatórias em pacientes submetidos à artroplastia de quadril por fraturas do colo do fêmur.

## Materiais e Métodos

### Desenho do Estudo e Seleção dos Pacientes

Este estudo retrospectivo foi realizado em nossa instituição entre 1° de abril de 2020 e 31 de março de 2025. Durante esse período, 1.119 pacientes foram submetidos à artroplastia de quadril por fraturas do colo do fêmur. Entre eles, 588 pacientes que receberam hastes femorais não cimentadas e foram submetidos à avaliação da DMO usando absorciometria de raios X de dupla energia ( *dual-energy X-ray absorptiometry* , DEXA, em inglês) até uma semana pós-operatória no quadril contralateral foram incluídos na análise. Pacientes com dados de DMO ausentes ou com informações clínicas incompletas foram excluídos.

### Coleta de Dados

A densidade mineral óssea foi medida usando DEXA com um escâner Lunar Prodigy (GE Healthcare). As regiões de interesse incluíram o colo femoral, o trocânter maior e a diáfise proximal. Os valores foram expressos como porcentagem da média de adultos jovens (YAM), com base em dados de referência japoneses de indivíduos de 20 a 39 anos.

As seguintes variáveis demográficas e clínicas foram coletadas dos prontuários: sexo, idade, lateralidade (direita ou esquerda), índice de massa corporal (IMC) e DMO. A DMO foi expressa como uma porcentagem da YAM, comumente usada no Japão como indicador da qualidade óssea.

### Classificação do Grupo

Os pacientes foram estratificados em 4 grupos conforme a classificação internacional do IMC (ponto de corte de 18,5 kg/m ^2^ ) e limiar de YAM (ponto de corte de 70%). Isso criou as seguintes categorias:

YAM ≥ 70% e IMC ≥ 18,5 kg/m ^2^ ;
YAM ≥ 70% e IMC < 18,5 kg/m ^2^ ;
YAM ≥ 70% e IMC ≥ 18,5 kg/m ^2^ ; e
YAM ≥ 70% e IMC < 18,5 kg/m ^2^ .

Além disso, os pacientes foram ainda categorizados em quatro grupos com base apenas nos níveis de YAM:

YAM ≥ 70%;
YAM 60–69%;
YAM 50–59%; e
YAM < 50%.

### Avaliação de Desfechos

O desfecho primário foi a ocorrência de fratura periprotética intraoperatória, que foi avaliada no intraoperatório e confirmada por achados intraoperatórios e por imagem pós-operatória.

### Análise Estatística

Além disso, entre 577 pacientes com dados completos de tempo operatório e perda de sangue, foram realizadas análises de subgrupos para comparar esses parâmetros operatórios entre pacientes com IMC ≥ 18,5 kg/m ^2^ e aqueles com IMC < 18,5 kg/m ^2^ . Os testes U de Mann-Whitney foram utilizados para avaliar a significância estatística.

A estatística descritiva foi utilizada para resumir as características do paciente. As taxas de fraturas intraoperatórias foram comparadas entre os grupos. A análise estratificada foi realizada para avaliar se o IMC modificou a associação entre o YAM e o risco de fratura. O teste exato de Fisher foi usado para calcular razões de chances (RCs) e valores de *p* . Um valor de *p* < 0,05 foi considerado estatisticamente significativo. As análises estatísticas foram realizadas com software padrão.

## Resultados

Um total de 588 pacientes foi incluído neste estudo ( Tabela 1 ). A idade média foi de 83,8 ± 8,1 anos, sendo 160 do sexo masculino e 428 do sexo feminino. O IMC médio foi de 20,19 ± 4,20 kg/m ^2^ , e o YAM médio foi de 62,4 ± 11,8%.

**Tabela 1:** Características basais estratificadas por YAM

Variável	YAM < 70%	YAM ≥ 70%
n	264	83
Idade média (anos)	84,6 ± 7,7	81,7 ± 8,0
Sexo feminino: n (%)	204 (77,3%)	54 (65,1%)
IMC médio (kg/m ^2^ )	19,50 ± 3,36	21,55 ± 3,57
YAM média (%)	57,4 ± 8,0	76,0 ± 7,4
Tempo operatório médio (minutos)	55,5 ± 18,1	58,7 ± 19,8
Perda de sangue média (mL)	134,7 ± 110,2	124,1 ± 72,5
Fratura:_n (%)	5 (1,9%)	3 (3,6%)

Abreviatura: YAM, *young adult mean* (“porcentagem média de adultos jovens”).

As características basais estratificadas por YAM são apresentadas na Tabela 1, e as estratificadas por categorias combinadas de YAM × IMC são apresentadas na Tabela 2 . Essas tabelas apresentam a distribuição detalhada das variáveis demográficas e operacionais entre os grupos analíticos.

**Tabela 2:** Características basais estratificadas por YAM e IMC

Variable	YAM < 70% e IMC < 18.5 kg/m ^2^	YAM < 70% e IMC ≥ 18.5 kg/m ^2^	YAM ≥ 70% e IMC < 18.5 kg/m ^2^	YAM ≥ 70% e IMC ≥ 18.5 kg/m ^2^
n	107	157	18	65
Idade média (anos)	85,5 ± 7,8	84,0 ± 7,6	79,5 ± 9,7	82,3 ± 7,5
Sexo feminino: n (%)	83 (77,6)	121 (77,1)	11 (61,1)	43 (66,2)
IMC médio (kg/m ^2^ )	16,66 ± 1,36	21,44 ± 2,91	16,37 ± 1,31	22,98 ± 2,49
YAM_média ± DP	55,0 ± 8,1	59,0 ± 7,6	73,8 ± 4,1	76,7 ± 8,0
Op tempo (min)_média ± DP	52,6 ± 15,8	57,5 ± 19,4	54,4 ± 15,9	59,8 ± 20,8
Perda de sangue (mL)_média ± DP	111,8 ± 85,7	150,7 ± 122,3	120,2 ± 60,9	125,2 ± 75,8
Fratura_n (%)	1 (0,9)	4 (2,5)	0 (0,0)	3 (4,6)

Abreviaturas: IMC, índice de massa corporal; YAM, *young adult mean* (“porcentagem média de adultos jovens”).

Os pacientes foram inicialmente classificados em 4 grupos com base na combinação de IMC (≥ 18,5 ou < 18,5 kg/m ^2^ ) e YAM (≥ 70% ou < 70%) ( Tabela 3 ). Fraturas periprotéticas intraoperatórias ocorreram em 5,36% dos pacientes com YAM ≥ 70% e IMC ≥ 18,5 kg/m ^2^ , 0,00% naqueles com YAM ≥ 70% e IMC < 18,5 kg/m ^2^ , 4,85% em pacientes com YAM < 70% e IMC ≥ 18,5 kg/m ^2^ e 7,10% naqueles com YAM < 70% e IMC < 18,5 kg/m ^2^ .

**Tabela 3:** Taxa de fratura intraperatória por YAM e IMC

Grupo	N	Fraturas (n)	Taxa de fratura (%)
YAM ≥ 70% e IMC ≥ 18,5 kg/m ^2^	112	6	5,36
YAM ≥ 70% e IMC < 18,5 kg/m ^2^	39	0	0,00
YAM < 70% e IMC ≥ 18,5 kg/m ^2^	268	13	4,85
YAM < 70% e IMC < 18,5 kg/m ^2^	169	12	7,10

Abreviaturas: IMC, índice de massa corporal; YAM, *young adult mean* (“porcentagem média de adultos jovens”).

A análise estratificada conforme o IMC mostrou que em pacientes com IMC ≥18,5 kg/m ^2^ , não foi observada associação significativa entre YAM e fratura intraoperatória (RC: 1,11; *p* = 0,80). Em contraste, em pacientes com IMC < 18,5 kg/m ^2^ , não foram observadas fraturas naqueles com YAM ≥ 70%, enquanto as fraturas ocorreram exclusivamente naqueles com YAM < 70% (RC :0,0; *p* = 0,128) ( Tabela 4 ).

**Tabela 4:** Análise estratificada conforme o índice de massa corporal (IMC)

Categoria IMC	Razões de chances	Valor de *p*
≥ 18,5 kg/m ^2^	1,11	0,80
< 18,5 kg/m ^2^	0,0	0,128

Além disso, quando os pacientes foram categorizados em quatro grupos com base apenas nos níveis de YAM (≥ 70%, 60–69%, 50–59% e < 50%), as taxas de fratura apresentaram tendência de aumento gradual: 3,97%, 5,24%, 5,88% e 6,58%, respectivamente. Embora essas diferenças não tenham atingido significância estatística, observou-se uma tendência dose-dependente ( Tabela 5 ). Além disso, entre os 577 pacientes para os quais os dados de tempo operatório e perda de sangue estavam disponíveis, os pacientes com IMC ≥ 18,5 kg/m ^2^ demonstraram tempo operatório significativamente maior (60,5 ± 20,9 minutos versus 55,3 ± 16,8 minutos; *p* = 0,006) e maior perda de sangue (133,5 ± 100,4 mL versus 109,5 ± 76,4 mL; *p* = 0,004) em comparação com aqueles com IMC < 18,5 kg/m ^2^ ( Tabela 6 ).

**Tabela 5:** Taxa de fratura intraperatória por categoria YAM detalhada

Grupo YAM	N	Fraturas (n)	Taxa de fratura (%)
≥ 70%	151	6	3,97
60–69%	191	10	5,24
50–59%	170	10	5,88
< 50%	76	5	6,58

Abreviatura: YAM, *young adult mean* (“porcentagem média de adultos jovens”).

**Tabela 6:** Comparação entre o tempo operatório e a perda de sangue conforme o índice de massa corporal (IMC)

Categoria IMC	n (N = 577)	Tempo operatório (médio) (minutos)	Perda de sangue média) (mL)	Valor de p (tempo operatório)	Valor de *p* (perda de sangue)
≥ 18,5 kg/m ^2^	372	60,5 ± 20,9	133,5 ± 100,4	0,006	0,004
< 18,5 kg/m ^2^	205	55,3 ± 16,8	109,5 ± 76,4		

Além disso, a análise de regressão logística multivariada, ajustada por idade, sexo, tempo operatório e perda de sangue, não identificou nenhum preditor independente de fratura intraoperatória; as razões de chance ajustadas para IMC e YAM baixos não foram estatisticamente significativas, provavelmente devido ao pequeno número de eventos. O teste de interação (IMC < 18,5 kg/m ^2^ × YAM < 70%) também não atingiu significância estatística. A análise de potência post hoc indicou que a diferença observada (≈ 1% versus 0% de taxa de fratura no grupo de baixo peso) forneceu apenas ∼ 12% de potência, enquanto uma diferença clinicamente significativa (6% versus 0%) teria rendido ∼ 49% de potência. Portanto, a falta de significância estatística provavelmente reflete um poder limitado, em vez de ausência de associação. Os parâmetros operatórios diferiram significativamente entre os grupos de IMC: pacientes com IMC ≥ 18,5 kg/m ^2^ tiveram maior tempo operatório (59,2 ± 19,3 versus 54,4 ± 15,5 minutos; *p* = 0,009) e maior perda de sangue (144,3 ± 116,2 vs 125,0 ± 93,1 mL; *p* = 0,024).

## Discussão

No tratamento contemporâneo de fraturas proximais do fêmur, a intervenção cirúrgica precoce, realizada dentro de 48 horas, tem sido consistentemente associada a melhores resultados. Moja et al. 1 relataram uma RC combinada de 0,74 (IC95%: 0,67–0,81) para mortalidade quando a cirurgia foi realizada dentro de 48 horas em comparação com atrasos, com base em > 190 mil pacientes. Da mesma forma, outra revisão sistemática confirmou redução da mortalidade de 30 dias e intra-hospitalar com cirurgia precoce 2

A escolha do implante também influencia o risco de fratura intraoperatória. Meta-análises descobriram que as hastes cimentadas estão associadas a significativamente menos fraturas periprotéticas, tanto intraoperatórias quanto pós-operatórias, do que as opções não cimentadas. 3 Os dados do registro suportam uma redução relativa do risco de 59% com hastes cimentadas. 4

Em nosso estudo, observamos uma clara tendência inversa entre o YAM pré-operatório e o risco de fratura intraoperatória, especialmente em pacientes de baixo peso (IMC < 18,5 kg/m ^2^ ), com uma tendência sugestiva, embora não atinja significância estatística. Isso indica que a avaliação do YAM pode fornecer uma estratificação de risco valiosa entre pacientes com baixo IMC.

O IMC está rotineiramente disponível na prática clínica e a avaliação pré-operatória do YAM em pacientes com baixo peso pode orientar o planejamento cirúrgico e a pré-reabilitação, incluindo o tratamento precoce da osteoporose.

Em contraste, entre os pacientes com IMC ≥ 18,5 kg/m ^2^ , as taxas de fratura não diferiram por YAM. Notavelmente, nossa subanálise de 577 pacientes mostrou que pacientes com IMC mais alto tiveram tempos operatórios significativamente mais longos (60,5 ± 20,9 versus 55,3 ± 16,8 minutos; *p* = 0,006) e maior perda de sangue (133,5 ± 100,4 versus 109,5 ± 76,4 mL; *p* = 0,004) ( Tabela 5 ). Isso indica que a complexidade do procedimento, em vez da qualidade óssea, impulsiona o risco nesse grupo. Por outro lado, a exposição simplificada em pacientes magros pode levar a uma inserção da haste mais rápida, porém mais arriscada, em contextos de baixa qualidade óssea.

Dado que radiografias simples e IMC estão universalmente disponíveis, há potencial para modelos de previsão orientados por inteligência artificial (IA) para estimar o YAM e prever o risco de fratura, o que melhoraria o planejamento pré-operatório em pacientes frágeis.

Embora a principal comparação entre pacientes com IMC < 18,5 kg/m ^2^ e YAM < 70% não tenha alcançado significância estatística ( *p* = 0,128), a tendência divergente observada entre os grupos de baixo e de alto IMC em relação à densidade mineral óssea é clinicamente significativa. A falta de significância provavelmente se deve ao poder estatístico limitado, particularmente porque o subgrupo de pacientes com IMC < 18,5 kg/m ^2^ e YAM ≥ 70% foi muito pequeno em nossa coorte. Isso destaca a necessidade de estudos multicêntricos maiores ou de análises agrupadas para confirmar e fortalecer essas descobertas preliminares.

Além disso, uma grande coorte recente demonstrou que a associação entre a DMO total do quadril com fraturas de quadril incidentes pode ser mais fraca entre indivíduos com baixo peso do que entre aqueles com IMC mais alto, apoiando nossos achados quanto ao aumento da vulnerabilidade à fratura em pacientes com baixo IMC e YAM. 5 Uma análise prospectiva em pacientes diabéticos confirmou, de forma semelhante, o baixo IMC como um fator de risco independente e poderoso para fraturas. 6 Revisões sistemáticas também desafiaram a noção de efeito protetor de que a obesidade é um fator de risco para fraturas de baixo trauma, enfatizando a complexidade das relações entre o IMC e as fraturas. 7 Finalmente, dados recentes sugerem que a saúde óssea subótima—incluindo baixa DMO e osteoporose—está significativamente associada as fraturas periprotéticas e a resultados ruins de artroplastia de quadril. 8 9

## Limitações

- Este foi um estudo retrospectivo, unicêntrico, realizado em nossa instituição.
- Apesar de observar tendências consistentes, as limitações do tamanho da amostra podem ter reduzido o poder estatístico. É necessária uma validação mais ampla e multicêntrica.
- Embora nossa instituição utilize predominantemente hemiartroplastia não cimentada, é possível uma mudança para hastes cimentadas. A fixação sem cimento continua a ser necessária em pacientes selecionados, e a validação externa em coortes variadas é necessária.

## Conclusão

Na hemiartroplastia não cimentada para fraturas do colo do fêmur entre pacientes com IMC < 18,5 kg/m ^2^ , a avaliação pré-operatória do YAM pode ajudar a reduzir o risco de fratura periprotética intraoperatória, destacando a importância da avaliação da qualidade óssea no planejamento cirúrgico de pacientes com baixo peso.
